# Evaluation of Walk Across Texas! – a web-based community physical activity program

**DOI:** 10.1186/s12889-019-7918-3

**Published:** 2019-11-28

**Authors:** Mark D. Faries, Michael L. Lopez, Ethan Faries, Kristen Keenan, Stephen D. Green

**Affiliations:** 1 Family & Community Health, Texas A&M AgriLife Extension Service, College Station, TX USA; 2 School of Public Health, Texas A&M Health Science Center, College Station, TX USA; 3 Tulane University, New Orleans, LA USA

**Keywords:** Physical activity, Walk Across Texas!, WAT!, Online, Community, Walking, Web, Team

## Abstract

**Background:**

In response to the chronic disease burden, web- and community-based programs have the potential to address targeted behaviors, such as physical activity (PA), using a novel approach with large audiences. The purpose of this study was to preliminarily evaluate an established team centered, web-based community PA program in Texas.

**Methods:**

Walk Across Texas! (WAT!) is an eight-week community program delivered through a web-based platform to help people of various ages and abilities establish the habit of regular PA. Teams are challenged to walk a minimum of 832 miles. Changes in self-reported PA (miles/week; days/week) and leisure-time sitting (hours/day) were examined from 11,116 adult participants who participated in the program in 2016. Further analysis determined changes in physical activity (miles/week) between groups of pre-program assessment self-reported physical activity levels (0, 1–2, 3–4, or 5–7 days/week). Statistical analysis included paired-sample t-tests, repeated measures ANOVA and participant descriptors for PA change.

**Results:**

Overall, mean changes in PA in all variables were statistically significant (*p* < .001), with the largest, clinically significant changes in submitted miles/week (mean increase of 4.89 ± 20.92). Self-reported PA increased 0.63 ± 2.89 days/week, while leisure-time sitting decreased less than 1 h per day (0.87 ± 1.86 h/day). All sub-groups (*inactive, low active, active, high active* at pre-program assessment) increased in self-reported miles per week, on average. Both the *inactive* and *low-active* groups experienced a statistically significant increase in mileage from week 1 to week 8 (5.48 miles/week or 12,330 steps /week, and 3.91 miles/week or 8797 steps /week, respectively).

**Conclusions:**

The results provide initial support for the effectiveness of WAT! to initially increase and maintain moderate levels of PA of participants over 8-weeks, even in inactive or low-active participants. Descriptor variables were unable to differentiate between those who increased PA and those who did not. However; the results provide a canvas for future research questions regarding PA enhancement within a team-centered, web-based approach.

## Background

Insufficient physical activity has been highlighted as a leading behavioral risk factor for mortality and disability in the United States behind poor dietary intake and smoking [1, 2]. According to data provided by the Centers for Disease Control and Prevention (CDC), however, only 1 in 5 adults meet physical activity guidelines, with those living in the South being less active than other parts of the United States [3]. Texas is no exception. According to the Behavioral Risk Factor Surveillance System, in 2015, less than 20% of Texas adults participated in enough aerobic and muscle strengthening exercise to meet the guidelines [4].

Interventions targeting physical activity have shown statistically significant effectiveness regarding behavior change and maintenance [5]. While these results are encouraging, effects are modest and difficult to determine the clinical significance [5, 6]. Interventional strategies used to address physical inactivity vary, however community-based approaches have the potential to reach large audiences. Community intervention locations include geographical boundaries (i.e. county) and settings that engage target populations (i.e. neighborhood, school, worksite, etc.) [7]. To reach populations within defined communities, web-based efforts can be low cost and effective [8].

### Walk Across Texas!

To promote increased physical activity, the Texas A&M AgriLife Extension Service [9] developed Walk Across Texas! (WAT!), an 8-week, web-based community physical activity program that utilizes a team approach to engage youth and adult participants. Founded in 1996, WAT! challenges teams consisting of up to eight members to walk a minimum of 832 miles (equivalent to virtually walking across the state of Texas) over the duration of the program. Participants track the amount of mileage walked each week, and either submit their results online, or send their weekly mileage logs to a Team Captain or Site Manager who then submits the information online, on behalf of team members. Although WAT! focuses primarily on walking as the means to achieve program goals, a program modification allows any activity to be counted towards individual and team mileage. A ‘Mileage Equivalence Calculator’ was developed using selected activities found in the Compendium of Physical Activities (https://sites.google.com/site/compendiumofphysicalactivities/home). Participation has continuously increased, and from 2012 to 2018, approximately 235,000 adults and youth have registered for the program from communities across Texas.

### Needed research

To date, limited research has been conducted to evaluate the effectiveness of community- and web-based physical activity programs in applied settings. Results from the few published studies reveal wide variability across programs, including program duration (2 to 11 weeks), physical activity outcomes, and measurements used in data collection [10–13]. Subsequently, such programs have demonstrated mixed results in terms of their impact on physical activity behavior and generalizability to the larger population [14]. Thus, more research is warranted with larger sample sizes containing in-depth descriptions of program details and implementation plans [15]. WAT! provides such an opportunity, with a reported pool of over 35,000 adult and youth participants in 2016.

In addition, despite its 20-year history of successful implementation, the WAT! program has never been formally evaluated using more rigorous methods of analysis. Therefore, the purpose of this study was to provide a preliminary evaluation of the impact of the WAT! program on participants’ self-reported physical activity using available data from 2016 adult participants. The primary aims were as follows:

1. Assess the overall change from week 1 to week 8 in physical activity in participants’ self-reported miles/week, as well as pre/post-program leisure-time physical activity (days/week), and pre/post-program leisure-time sitting (hours/day). This aim provides insight into the program’s effectiveness.
2. Determine any changes in physical activity (miles/week) between groups of pre-program assessment self-reported physical activity (0, 1–2, 3–4, or 5–7 days/week). This aim provides insight into the effectiveness of the program for participants of varying activity levels.
3. Examine variation in descriptive measures between participants who either had *no change*, an *increase*, or a *decrease* in mileage from week 1 to week 8. This aim provides insight into any potential descriptors that might relate to increased physical activity as a result of the program.

## Methods

### Participants

The data set included 11,116 adult participants (≥ 18 years of age) who entered their mileage for both week 1 and week 8. A total of 5803 participants were excluded due to incomplete data or a self-reported age of less than 18 years. Finally, 5 participants were determined to be outliers in mileage entered (135 to 250 miles; 3 SDs above the mean), and were removed, resulting in the current sample size of 11,116. Descriptive data are provided in Table 1.

**Table 1:** Participant characteristics, overall and within mile change groups (week 8 minus week 1)

		Mile Change Groups		
Variable	Overall (*N* = 11,116)	No Change (*n* = 606)	Increase (*n* = 6452)	Decrease (*n* = 4058)
Age (years)	44.89 ± 14.06	47.45 ± 17.07	44.62 ± 13.85	44.95 ± 13.85
Gender (%)^a^				
Women	76.6	73.4	76.8	76.9
Men	23.3	26.6	23.2	23.1
Race (%)^a^				
Anglo	70.3	68.2	69.4	72.0
Hispanic	15.9	15.5	16.0	15.9
African American	8.5	8.9	9.1	7.5
Native American	1.2	2.0	1.4	0.8
Multiracial	1.7	2.2	1.9	1.5
Asian	1.6	0.8	1.6	1.7
Hawaiian	0.1	1.2	0.0	0.1
Physical Activity (days/week)	4.17 ± 2.24	4.00 ± 2.31	4.15 ± 2.26	4.24 ± 2.20
Leisure Sitting Time (%)^a^				
< 1 h/day	25.4	41.6	25.6	22.6
1–3 h/day	52.9	42.1	52.7	55.0
≥ 4 h/day	21.7	16.3	21.7	22.4
Activity Type (%)^b^				
Other Activity	94.0	95.4	93.4	94.8
Walk	20.5	16.0	22.0	18.9
Run	5.2	2.1	5.4	5.2
Bike	3.4	2.3	3.8	3.0
Swim	1.2	0.7	1.3	1.1
Activity Location (%)^b^				
Other Location	95.2	98.2	94.5	95.8
Neighborhood	48.9	50.0	48.7	49.1
Park	32.9	28.2	33.4	32.8
Worksite	29.8	23.3	29.8	30.8
Gym	28.9	31.4	29.2	28.0
Home	23.0	21.1	23.1	23.2
Track	10.7	11.4	11.2	9.8
Mall	5.3	5.8	5.1	5.7
Reason for Participation (%)^a^				
Personal Health	33.4	34.0	33.9	32.4
Employee Wellness Program	21.9	13.7	22.0	22.9
WAT! Challenge	18.3	12.5	17.2	20.9
Support Friend/Family	7.1	7.3	7.2	6.9
School Event	3.3	2.0	3.6	3.2

^a^Cumulative percentage; ^b^Percent of responses (i.e. those answering that item)

### Measures

All measures and evaluation criteria were developed for internal evaluation and program development purposes. Data was collected from online program registrations that occurred between January – November 2016. These measures are detailed below.

#### Personal descriptives

Self-reported age was assessed in years, and the assessment for race/ethnicity provided 7 different choice options (see Table 1). Self-reported weight was asked at the pre-program assessment; however, only 3% (*n* = 461) responded to this item, thus it was excluded from the present analysis. Height was not collected. Participants were also asked their reasons for participation and were instructed to choose one from the following list: WAT! challenge, personal health, employee wellness program, support a friend or family, or school event.

#### Physical activity

Participant mileage, the primary measurement of physical activity during the 8-week program, was entered online through the WAT! website. Team members were instructed to submit weekly mileage totals to the Team Captain for entry into the online system or could submit their own results directly in a team member account. For individuals who elected not to participate on a team, but as “solo walkers,” mileage was entered online by the individuals themselves. For the present study, mileage entered at week 1 and week 8 were analyzed.

Both pre- and post-program assessments were used to clarify physical activity behavior in the sample. For physical activity level, participants were asked, “*During the past 7 days, on how many days were you physically active for at least 30 minutes per day? Add up all the time spent in any activity that increased your heart rate, and made you breathe hard some of the time,”* [16, 17]. Responses ranged from 0 to 7 days. Using the pre-program assessment, participants were assigned to a group that represented their pre-program physical activity level (0, 1–2, 3–4 or 5–7 days/week; see *aim 2*).

For sitting time, participants were asked, *“On most days, how many hours per day do you spend sitting while at home and/or during leisure time. This may include time spent visiting friends, reading, or watching television,”* [17]. Responses were less than 1 h, 1 h, 2 h, 3 h and 4 h or more.

In addition to their activity level, participants were asked about their activity type(s) and activity location(s). For *type*, they chose from the following list: walk, run, bike, swim, or other activity. For *location*, they could choose from the following list: home, neighborhood, park, worksite, gym, track, mall, or other location. For both items, multiple responses were allowed.

### Statistical analysis

For *aim 1*, three separate paired-sample t-tests were used to determine if statistically significant changes occurred in overall physical activity based on self-reported mileage, physical activity level (in days) and leisure-time sitting (in hours). For *aim 2*, a 2 × 4 (time by group) repeated-measures factorial ANOVA was used to examine differences in the week 1 to week 8 change in self-reported miles between groups derived from self-reported pre-program physical activity level. For *aim 3*, means and standard deviations or frequency (percentage of participants (%)) of each variable were calculated for each mileage change group (i.e. no change, increase, decrease). The alpha-criterion was set at *α* = 0.05 for all analyses.

## Results

### Participants

On average, participants were middle-aged (44.89 ± 14.06 years of age), and mostly self-reported Anglo/Caucasian women (Table 1). Most participants were part of a team (98%), while 1.3% participated as an individual, and 0.7% were undeclared. Before beginning the program, participants were moderately physically active, reporting an average of 4 days per week of activity for at least 30 min per day. A majority (77%) reported that they engaged in 3–7 days/week of physical activity, with 10% reporting 0 days/week. Also, nearly 53% of participants reported sitting only 1–3 h per day, while only 22% indicated that they sit ≥4 h per day.

Generally, personal health, an employee wellness program, and a WAT! challenge in their community accounted for 60% of reasons for participation. The leading self-reported activity locations during the program for 93% of participants (*n* = 10,331) were in one’s neighborhood (49%), followed by the park (33%), worksite (30%), gym (29%), or at one’s home (23%). In addition, 95% of these participants noted other locations for their physical activity. Only 22% of the sample (*n* = 2426) reported their activity type. Of those who responded, walking (21%) was the most common self-reported type of activity.

### Change in physical activity (overall)

Table 2 provides changes in physical activity from week 1 and week 8 for self-reported miles/week, days/week of physical activity of at least 30 min per day, and leisure-time sitting in hours/day. Statistically significant improvements (*p* < .001) occurred on all three variables, with the largest, clinically significant results shown in miles/week (average increase of 4.89 ± 20.92 miles/week). Participants’ self-reported physical activity increase was 0.63 ± 2.89 days/week, while leisure-time sitting decreased less than 1 h per day (0.87 ± 1.86).

**Table 2:** Self-reported physical activity for overall and sub-samples (mean ± standard deviation)

	Pre-Program	Post-Program	*t*	*p*
Physical Activity (days/week)^a^	4.17 ± 2.24	4.80 ± 2.27	−22.94	< 0.0001
0 days/week	10.0%	11.1%		
1–2 days/week	13.3%	4.7%		
3–4 days/week	27.9%	19.5%		
5–7 days/week	48.8%	64.7%		
Sitting Time (hours/day)^b^	2.02 ± 1.48	1.16 ± 1.38	48.92	< 0.0001
	Week 1	Week 8	*t*	*p*
Miles/week	26.66 ± 20.69	31.54 ± 26.59	−24.62	< 0.0001
*Gender*				
Women	25.68 ± 19.49	30.51 ± 25.13	−22.45	< 0.0001
Men	29.88 ± 23.98	34.97 ± 30.68	−10.73	< 0.0001
*Age Group*				
18–29 years of age	26.85 ± 21.67	31.78 ± 27.81	−9.38	< 0.0001
30–49 years of age	27.44 ± 20.52	32.45 ± 26.51	−16.80	< 0.0001
50–64 years of age	26.93 ± 20.32	31.86 ± 26.43	−13.52	< 0.0001
≥ 65 years of age	21.20 ± 20.47	25.20 ± 26.60	−7.54	< 0.0001
*Race/Ethnicity Group*				
Anglo	26.99 ± 20.81	31.62 ± 26.54	−19.70	< 0.0001
Hispanic	27.73 ± 22.41	32.31 ± 27.90	−8.84	< 0.0001
African American	23.15 ± 16.47	30.12 ± 24.05*	−10.72	< 0.0001
Other	24.79 ± 19.25	30.58 ± 27.09	−6.60	< 0.0001

^a^Self-reported at least 30 min per day in the past 7 days

^b^Hours sitting during leisure time on most days

*Significantly different than Anglo and Hispanic groups (*p* < 0.01)

### Change in physical activity (by pre-program activity group)

**Fig. 1:**
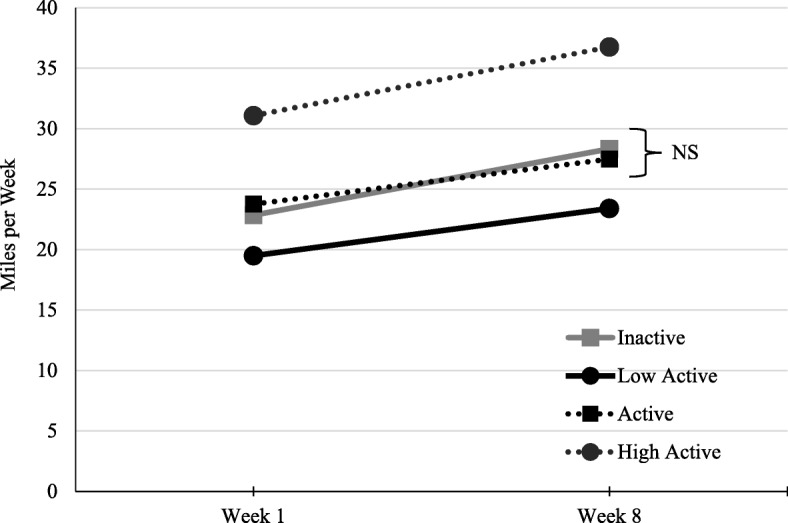
Mean self-reported mileage at week 1 and week 8 by pre-program physical activity groups. NS = non-significant difference. All other groups were significantly different. *Note*. Pre-program physical activity groups were based on self-reported days/week of moderate-intensity activity: Inactive (0 days/week), Low Active (1–2 days/week), Active (3–4 days/week), High Active (5–7 days/week)

Based on their pre-program physical activity level (days/week; Fig. 1), participants were divided into 4 groups: Inactive (0 days/week), Low Active (1–2 days/week), Active (3–4 days/week) and High Active (5–7 days per week). A statistically significant main effect was found for change in self-reported miles (Wilks’ Lambda = 0.97; *F* (1,11,112) = 389.36), as well as a significant interaction effect between groups (Wilks’ Lambda = 0.99; *F* (3,11,112) = 7.12). The effect sizes for both the main effect (η_p_^2^ = 0.034) and interaction effect (η_p_^2^ = 0.002) were small; however, the Tukey post-hoc revealed that all group changes were significantly different from each other (*p*s < 0.00), with the only exception being a non-significant difference between Inactive and Active groups (*p* > 0.05).

### Change in physical activity (by gender, age, race/ethnicity)

Changes in physical activity (self-reported miles) from week 1 to week 8 were also analyzed by self-reported gender, age and race/ethnicity groups (Table 2). As expected, a statistically significant main effect was confirmed in self-reported miles across all three analyses (Wilks’ Lambda = 0.96–0.97; F = 318.65–446.38).

No significant interaction effect between genders in mileage change was found (Wilks’ Lambda = 1.00; *F* (3,11,111) = 0.32). For self-reported age, participants were divided into 4 groups: 18–29 years (15.0%), 30–49 years (46.3%), 50–64 years (30.1%) and ≥ 65 years of age (8.6%). No significant interaction effect between age groups was found (Wilks’ Lambda = 1.00; *F* (3,11,109) = 0.64). A weak, yet significant interaction effect was found between race/ethnicity groups (Wilks’ Lambda = 9.99; *F* (3,11,112) = 4.00; η_p_^2^ = 0.001), which were divided into 4 groups: Anglo (70.3%), Hispanic (15.9%), African American (8.5%) and Other (5.2%). Specifically, participants self-reporting as African American slightly increased mileage over the 8 weeks more so than those self-reporting as Anglo (*p* < 0.01) and Hispanic (*p* = 0.001).

### Participant descriptors and physical activity change

Analysis of descriptor variables between groups who had no change, an increase, or a decrease in miles per week from week 1 to week 8 (Table 1) revealed no obvious and consistent, clinically significant differences. In other words, each group was of similar age, gender and race distribution, initial physical activity level, activity type or location, and reason for participation.

## Discussion

The primary objective of the present research was to evaluate an established 8-week community- and web-based physical activity program in Texas, using available data from 2016. Generally speaking, this preliminary research supports the effectiveness of the 8-week WAT! program to attract participants from all physical activity levels, and to help inactive/low active participants become and remain physically active over the course of the program.

### Change in physical activity

First, we examined the overall change from week 1 to week 8 in self-reported physical activity, and if such changes varied by pre-program physical activity levels. On average, participants achieved nearly 27 miles of self-reported physical activity at week 1, and progressed to just over 31 miles, by week 8. As mentioned, the largest, clinically significant results were shown in miles, with an average increase of 4.89 ± 20.92 miles per week. Using the estimate of 2250 steps per mile at a moderate intensity of 3 mph, [18] the average increase of 4.89 miles per week equates to an additional 11,002 steps/week (1571 steps/day). Of particular interest, both the inactive and low-active groups experienced a statistically significant increase in mileage from week 1 to week 8 (5.48 miles/week or 12,330 steps /week, and 3.91 miles/week or 8797 steps /week, respectively).

It should be noted, however, that these are estimates to help conceptualize mileage within the program. With the option to utilize a developed physical activity calculator (based on MET values), a variety of physical activities could have accounted for this mileage ‘walked’. Regardless, on average, the program was effective in improving self-selected physical activity, while highlighting the large variability in physical activity change within a large sample of participants.

With the unique mechanism of online data collection and mileage calculation, it is difficult to compare the present results to the limited previous research on web-based group physical activity programs. A review of internet-based physical activity interventions showed that just over 60% of studies reported a significant improvement in physical activity, while approximately 37% reported null outcomes [11]. In addition, 61% of studies using subjective measures of physical activity, such as those used in the WAT! program, found significant improvements. Thus, these preliminary results support WAT! to be aligned with other programs able to achieve improvement in physical activity during program implementation.

We were unable to specifically locate and operationalize the reasons that this program was effective in helping participants achieve an initial bump in and maintenance of physical activity; however, we might speculate to guide future research and program development. First, 98% of participants in this sample were a part of a team, which could enhance effectiveness. Social support could be considered here; however, the relationship between social support and future physical activity, although positive, appears to be inconsistent [19]. The team-based approach could hold differing effects than simply providing social support. For example, social comparison, as experienced in team-based settings, might be more effective for increasing physical activity than social support [20, 21].

Also, with respect to all variables, there was a wide variation in the physical activity levels and change over the course of the program. The average miles/week increase was 4.89, but the average distance from this mean was 20.92 miles. The minimum change in mileage from week 1 to week 8 was − 195.80 miles/week and the maximum change was 225.00 miles/week. Approximately 64% of the sample reported a change from − 10 to + 10 miles/week (including those submitting 0 miles/week, *n* = 606), which provide further support for the general improvement in physical activity, on average, across the sample. However, these findings highlight several factors that could possibly account for such variation, and research into these factors would be highly fruitful and informative for program development.

From a programmatic standpoint, we were also interested in changes in physical activity based on pre-program physical activity level (i.e., before starting the program). Such information would provide points of interest for how to improve the program in the future.

For pre-activity level, we were most interested in those who were ‘inactive’ or ‘low active,’ as these participants are a target population for improving physical activity levels. We note that only 23% of the sample fell into these two groups, while 77% were self-reported achieving between 3 and 7 days per week before starting the program. Nearly half of participants (49%), self-reported meeting physical activity guidelines of 30-min per day of a moderate intensity activity on at least 5 days per week. It should be noted, however, that after the program the proportion of those self-reporting meeting the guidelines increased to 65% (see Table 2). These findings have implications for future efforts to market the program in such a way to enhance participation from low- or non-active individuals.

The changes in self-reported mileage from week 1 to week 8 between groups divided by their pre-program physical activity levels are shown in Fig. 1. A unique phenomenon occurred, in that all groups had similar starting points of self-reported mileage at week 1, revealing that even those classified as ‘inactive’ or ‘low-active’ (≤ 2 days/week) before beginning the program self-reported achieving moderate levels of physical activity within the first week of participation, and experienced a similar change in self-reported miles per week through week 8. In one aspect, these results are encouraging, as even those who were inactive or low active (i.e., not meeting physical activity guidelines) before the program started (i.e., ‘pre-program’) could reach and maintain moderate levels of physical activity during the 8 weeks, as measured by their self-reported mileage. Thus, WAT! was able to help even the lowest of active individuals, rapidly achieve higher levels of physical activity. While these results could be attributed to the social aspects, as previously mentioned, it is unclear why this phenomenon occurred. Such results might be unique, and further evaluation following the 8-week program could confirm any stability of physical activity maintenance.

By suggestion of reviewers, we also conducted post-hoc analyses of differences in changes in self-reported mileage between self-reported gender, age groups and race/ethnicity groups (Table 2). Generally, we found that while there were significant, albeit weak, main effects, of an average increase in mileage, no differences were found between genders or age groups. There was a significant, although weak difference between those self-reporting as African American, whereby they slightly increased in mileage from week 1 to week 8 more so than those self-reporting as Anglo or Hispanic. In summary, these results support the WAT! program's ability to not only attract a diverse group of participants, but also positively impact their physical activity levels.

Finally, the analysis of descriptor variables between groups who had *no change*, an *increase*, or a *decrease* in miles per week from week 1 to week 8 (Table 1) revealed no glaring and consistent clinically significant differences. From a program development standpoint, few suggestions can then be made on what participant characteristics might relate to or possibly predict an increase in mileage (or maintain) over the course of the program. Each group was of similar age, gender and race distribution, initial physical activity level, activity type or location, and reason for participation. These results suggest that other factors exist to help explain the variation in physical activity across the 8 weeks, of which future research can elucidate.

### Limitations

Several limitations should be noted. First, this research study was an observational, post-hoc analysis of a long-standing, active program, which had determined its design and measures beforehand without research evaluation input. All measures were self-reported, and items were developed by the program team for internal use. While the measures used to assess pre and post physical activity levels were slightly modified versions of widely-used validated items, they were not fully validated prior to this study. As a result, the accuracy of the self-reported physical activity data when compared to actual activity levels could be called into question.

However, the same measures were used at each measurement time point, providing initial confidence for assessing change in physical activity over time. Future programmatic research would benefit from the usage of known measures for all assessments that have been deemed both valid and reliable. Similarly, the novelty of the Mileage Equivalence Calculator makes it difficult to compare the present results to other studies, and highlights potential variation in self-reported physical activity that is poorly understood. A strength is that the calculator allows for individuals to incorporate many activities, beyond walking, into their daily and weekly mileage accumulation. This autonomy could allow for enhanced results, but is speculative at this time. The data is also somewhat limited due to only evaluating change from week 1 to week 8 with no assessments in-between. With the stability of self-reported physical activity from week 1 to week 8, it would be interesting and informative to explore what variation occurred, if any, during weeks 2–7.

As for the large sample, which is a study strength, participants were generally fairly active, averaging around 4 days (30 min or more) of moderate to intense physical activity per week. Due to the sample composition of predominately middle-aged, Anglo/Caucasian females, caution is suggested in extrapolating these results too far beyond the sample demographics or specific programmatic features. Future research should evaluate the WAT! program in a more stringent randomized-control trial with a more diverse sample and theoretically-based measures to assess novel factors that could explain the variation in outcomes across such a large sample.

## Conclusion

The purpose of this study was to evaluate a large and established 8-week team centered, community web-based physical activity program in Texas (Walk Across Texas!) using available data from 2016. With a sample of over 11,000 adult participants, these preliminary results support the effectiveness of the 8-week WAT! program to attract participants from all physical activity levels, and to help inactive/low active participants become and remain physically active. On average, self-reported mileage from various physical activities increased nearly 5 miles per week, with large variation across the sample (± 21 miles per week). Also, this increase and maintenance of physical activity appeared to be consistent across all pre-program physical activity levels, from inactive to high active individuals, as well as across demographic variables. Descriptor variables were unable to differentiate between those who increased physical activity and those who did not; however; the results provide a canvas for future research questions regarding physical activity enhancement within a team-centered, web-based approach.

## Data Availability

The dataset supporting the conclusions of this article is available upon request by contacting Mark D. Faries at Mark.Faries@ag.tamu.edu

## Abbreviations

BRFSS: Behavioral risk factor surveillance system
CDC: Centers for disease control and prevention
WAT!: Walk Across Texas!
